# Attitudes and beliefs towards early ART initiation in MSM with primary HIV infection

**DOI:** 10.7448/IAS.17.4.19732

**Published:** 2014-11-02

**Authors:** Victoria Parsons, Kholoud Porter, Richard Gilson, Graham Hart

**Affiliations:** 1 Department of Infection and Population Health, University College London, London, UK; 2 MRC CTU, University College London, London, UK

## Abstract

**Introduction:**

ART initiation in primary HIV infection (PHI) could reduce risk of transmission to sexual partners at a time of high viraemia, although health benefit for the individual remains unknown. We examined attitudes to early ART and associated beliefs in men who have sex with men (MSM) with PHI.

**Materials and Methods:**

Semi-structured face-to-face in-depth interviews were conducted with 13 MSM aged ≥16 years attending a central London HIV clinic, within 12 months of date of estimated HIV seroconversion. Audio recordings of interviews were transcribed verbatim and analyzed thematically.

**Results:**

Median age was 33 years (range 22–47), majority were white British (n=8), educated to university level (n=11) and were not on ART (n=10). Great diversity in ART knowledge and expectations around starting were observed, with some men assuming they would be prescribed ART immediately upon diagnosis. Deferral until CD4<350 came as a surprise and counterintuitive when put into the context of treating other diseases. For many, the decision to start ART was a balance of current and future health and quality of life. Fear of side effects was prevalent, with many believing them inevitable and a reason to avoid early ART. A perceived lack of “good quality” evidence showing a health benefit of early ART caused confusion. Avoiding the decision to start or deferring to their HIV clinician was common, however reported clinicians’ views also varied. Some men voiced a desire to be proactive and start early ART to control viral replication. In these cases men also reported a belief that ART could be temporary as they expected a cure in their lifetime. Men commonly described feeling “infected” and reducing this infectiousness was seen as a major benefit of ART; not purely to reduce the risk of transmission to sexual partners but to facilitate disclosure to partners, reduce anxiety and guilt and restore sexual confidence commonly lost after HIV diagnosis. Having a long-term HIV-negative partner was a strong facilitator to starting ART to reduce transmission in the absence of good evidence of individual health benefit.

**Conclusions:**

Factors involved in the decision to start ART in PHI were complex. Uncertainty over individual health benefits in conjunction with fear of toxicities were barriers to starting ART early. By contrast ART was seen as a facilitator to disclosure, and as a way to limit the consequences of infection until a cure is found.

